# Supplementary material for: Microbial community in buckwheat rhizosphere with different nitrogen application rates
Source: PeerJ. 2023 Jun 21;11:e15514. doi: 10.7717/peerj.15514 (PMC10290450; doi:10.7717/peerj.15514)
Supplement: Supplemental Information 1 [file peerj-11-15514-s001.docx]

Table S1 Chemical properties of the samples

| Treatments | pH | Available nitrogen (mg/kg) | Total nitrogen (g/kg) |
| --- | --- | --- | --- |
| N90 | 7.86±0.05 ^a^ | 2.01±0.03 | 1.35±0.08 |
| N120 | 7.85±0.02 | 2.03±0.05 | 1.38±0.06 |
| N150 | 7.88±0.04 | 1.98±0.04 | 1.36±0.10 |

^a^ Two-tailed t-test was used to analyze the variances among groups. The results showed that there was no significant difference (df=2, P>0.05) in all of the three indices.

Table S2 Valid reads obtained from the samples

| Samples | Raw reads | Ratio of valid reads | Valid reads |
| --- | --- | --- | --- |
| N90_1 | 85436866 | 99.63 | 85120749.6 |
| N90_2 | 100730128 | 99.68 | 100407791.6 |
| N90_3 | 86890742 | 99.67 | 86604002.55 |
| N120_1 | 95538976 | 99.65 | 95204589.58 |
| N120_2 | 92548524 | 99.65 | 92224604.17 |
| N120_3 | 88939526 | 99.6 | 88583767.9 |
| N150_1 | 86687642 | 99.68 | 86410241.55 |
| N150_2 | 94703168 | 99.62 | 94343295.96 |
| N150_3 | 83325472 | 99.61 | 83000502.66 |

Table S3 Alpha diversity indexes of soil microbes

| Treatments | Simpson | Chao1 (×10^4^) | ACE (×10^4^) | Shannon |
| --- | --- | --- | --- | --- |
| N90 | 0.62±0.03 ^a^ | 2.79±0.02 | 2.76±0.02 | 4.43±0.21 |
| N120 | 0.59±0.02 | 2.81±0.02 | 2.79±0.02 | 4.09±0.23 |
| N150 | 0.61±0.03 | 2.80±0.02 | 2.78±0.02 | 4.36±0.23 |

^a^ Two-tailed t-test was used to analyze the variances among groups. There was no significant difference (df=2, P>0.05) in all of the three indices.

Table S4 Numbers of functions annotated by genes with significantly different relative abundance

| Group pairs | KO | eggNOG | CAZyme |
| --- | --- | --- | --- |
| N150-N120 | 0 | 0 | 0 |
| N150-N90 | 0 | 0 | 0 |
| N90-N120 | 0 | 2 | 0 |


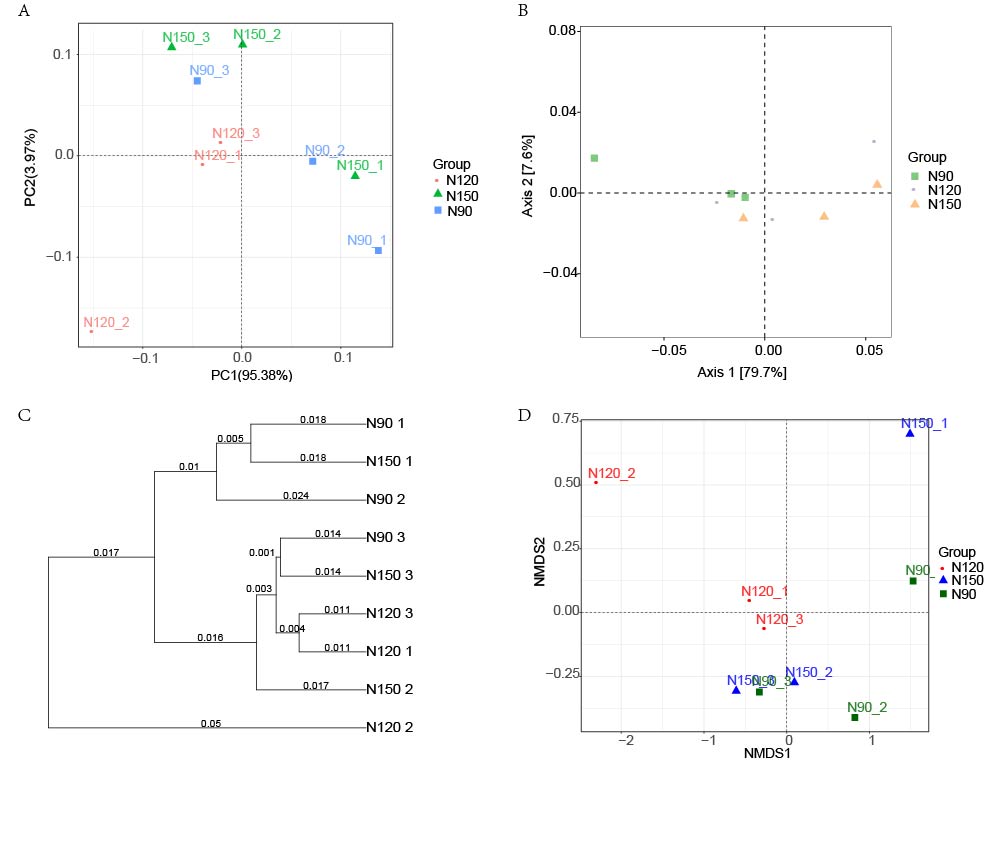


Figure S1 Beta diversity of microorganism in buckwheat rhizosphere. A. PCA analyze. B. Principal coordinates analysis (PCoA) analyze. C. Hierarchical clustering by unweighted pair-group method with arithmetic means (UPGMA). D. Nonmetric multidimensional scaling (NMSD) analyze.


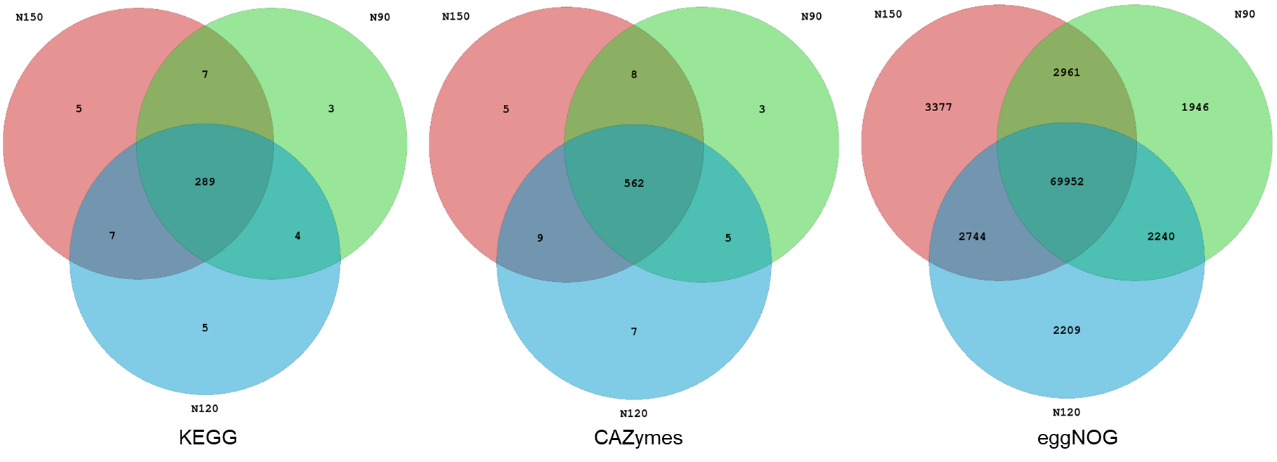


Figure S2 Microbial function numbers in rhizosphere of buckwheat with different nitrogen application rates.
